# Sequential Coordination between Lingual and Pharyngeal Pressures Produced during Dry Swallowing

**DOI:** 10.1155/2014/691352

**Published:** 2014-12-18

**Authors:** Jitsuro Yano, Yoichiro Aoyagi, Takahiro Ono, Kazuhiro Hori, Wakami Yamaguchi, Shigehiro Fujiwara, Isami Kumakura, Shogo Minagi, Akio Tsubahara

**Affiliations:** ^1^Department of Speech-Language-Hearing Therapy, Rehabilitation Center, Kawasaki Medical School Hospital, 577 Matsushima, Kurashiki, Okayama 701-0114, Japan; ^2^Department of Occlusal and Oral Functional Rehabilitation, Graduate School of Medicine, Dentistry and Pharmaceutical Sciences, Okayama University, 2-5-4 Shikata-cho, Kita-ku, Okayama 700-8525, Japan; ^3^Department of Rehabilitation Medicine, School of Medicine, Fujita Health University, 1-98 Dengakugakubo, Kutsukake, Toyoake, Aichi 470-1192, Japan; ^4^Department of Prosthodontics, Gerodontology and Oral Rehabilitation, Osaka University Graduate School of Dentistry, 1-8 Yamada-oka, Suita, Osaka 565-0871, Japan; ^5^Division of Dysphagia Rehabilitation, Niigata University Graduate School of Medical and Dental Sciences, 2-5274 Gakkocho-dori, Chuo-ku, Niigata 951-8514, Japan; ^6^Physiological Function Research Center, Kawasaki Medical School Hospital, 577 Matsushima, Kurashiki, Okayama 701-0114, Japan; ^7^Department of Sensory Science, Faculty of Health Science and Technology, Kawasaki University of Medical Welfare, 288 Matsushima, Kurashiki, Okayama 701-0193, Japan; ^8^Kawasaki University of Medical Welfare, 288 Matsushima, Kurashiki, Okayama 701-0193, Japan

## Abstract

The aim of this study was to investigate oropharyngeal pressure flow dynamics during dry swallowing in ten healthy subjects. Tongue pressure (TP) was measured using a sensor sheet system with five measuring points on the hard palate, and pharyngeal pressure (PP) was measured using a manometric catheter with four measuring points. The order and correlations of sequential events, such as onset, peak, and offset times of pressure production, at each pressure measuring point were analyzed on the synchronized waveforms. Onset of TP was earlier than that of PP. The peak of TP did not show significant differences with the onset of PP, and it was earlier than that of PP. There was no significant difference between the offset of TP and PP. The onset of PP was temporally time-locked to the peak of TP, and there was an especially strong correlation between the onset of PP and TP at the posterior-median part on the hard palate. The offset of PP was temporally time-locked to that of TP. These results could be interpreted as providing an explanation for the generation of oropharyngeal pressure flow to ensure efficient bolus transport and safe swallowing.

## 1. Introduction

During swallowing movements from the oral stage to the pharyngeal stage, following elevation of the tongue, a smooth transition of movement from laryngeal elevation to pharyngeal contraction and then to relaxation of the upper esophageal sphincter (UES) produces smooth food bolus propulsion [1–3]. In this process, tongue muscles and pharyngeal constrictors play crucial roles in bolus propulsion, and in order to smoothly expel the bolus from the oral cavity toward the pharynx, and from there to the esophagus, contraction is carried out with the appropriate timing, in the appropriate sequence, to produce swallowing pressure [4–9]. A failure of bolus propulsion results in residue in the oral cavity, pharyngeal residue, and laryngeal penetration, and aspiration is frequently seen, not only among patients with impaired swallowing, but among the elderly population as well [10–13]. In order to ascertain the condition of swallowing impairment more accurately, it is necessary to understand not only dysmobility of the individual organs involved in oral cavity and pharyngeal swallowing, but also the time-based coordination of the movements of the various organs.

To investigate the coordination of the oral cavity and pharyngeal organs when swallowing, videofluorography (VF) was first used to analyze morphological changes taking place over time in the oral cavity and pharyngeal organs, as well as changes in the bolus propulsion time, both quantitatively and qualitatively, on images [14–16]. Next, VF and manofluorography, which simultaneously measure the internal pressures of the pharynx and the esophagus, were used to clarify the relationships between the morphological changes in the pharynx and the flow of bolus propulsion and intraluminal pressure flow [17, 18]. There are numerous reports describing measurements of pharyngeal pressure, and the maximum pharyngeal pressure, pharyngeal pressure duration, and time that the UES is open have been analyzed [19–22]. Recently, there have been an increasing number of reports on pharyngeal pressure using high-resolution manometry (HRM), and the topography obtained using HRM has made it possible to ascertain the status of pressure being propagated from the upper pharynx to the esophagus [9, 23, 24].

In the past, the tongue-palate contacting pressure [20], which is the largest motive force sending the food bolus to the pharynx, was measured only at one location, using a balloon that was inserted into the oral cavity [25–27]. However, Hori et al. [6] developed an ultrathin sensor sheet capable of measuring the tongue-palate contact pressure (tongue pressure) at numerous points on the palate, and in addition to elucidating the onset order and pressure gradient of the pressure from the front to the back of the oral cavity in healthy subjects, they reported a certain degree of coordination between the onset of tongue pressure and hyoid movement at the time of swallowing in healthy subjects [7]. A correlation between abnormalities in the tongue pressure waveform seen in patients whose swallowing is impaired because of cerebrovascular disease, nerve or muscle disorders, or oral cavity cancer and attenuation of pressure and swallowing impairment has also been reported [28, 29].

Thus, there are numerous reports describing the production of tongue pressure and pharyngeal pressure when swallowing. However, no reports have described simultaneous measurements of the series of pressure flow dynamics of the tongue pressure and pharyngeal pressure from the oral cavity to the pharynx. If the temporal coordination taking place from the tongue pressure to the pharyngeal pressure to the opening of the esophageal orifice could be analyzed, it might be possible to analyze the pathophysiology of aspiration prior to and after swallowing, which is thought to occur if the coordination of pressure production breaks down, in greater detail. Given that, the authors analyzed the temporal coordination between the tongue pressure that occurs when swallowing saliva and the production of pharyngeal pressure from the oral cavity and pharynx, conducting simultaneous measurements using a tongue pressure sensor sheet and manometry. Based on the results, the mechanism at work in bolus propulsion was then considered.

## 2. Materials and Methods

### 2.1. Subjects

Ten healthy subjects (3 men and 7 women; age range, 21–27 years; mean age, 22.1 ± 2.1 years) without disturbances of mastication and deglutition, abnormalities in the number or position of teeth except for the third molar, history of orthodontic treatment and temporomandibular disorders, and abnormality in occlusion were included in this study. Written, informed consent was obtained from each subject after explaining the aim and methodology of the study. This study received approval by the ethics committee of the Kawasaki University of Medical Welfare.

### 2.2. Experimental Setting

Subjects were asked to sit on a chair with their head vertical to the floor. They were then asked to save saliva in an oral cavity and swallow saliva according to the examiner's instructions. They repeated dry swallowing 5 times with the tongue pressure sensor sheet on the hard palate and the manometric catheter through the pharynx. Details of the methodology of each measurement are described below. An adaptation period of 15 minutes was allowed before the measurements, and five minutes were left between each measurement for resting and rinsing the mouth.

**Figure 1:**
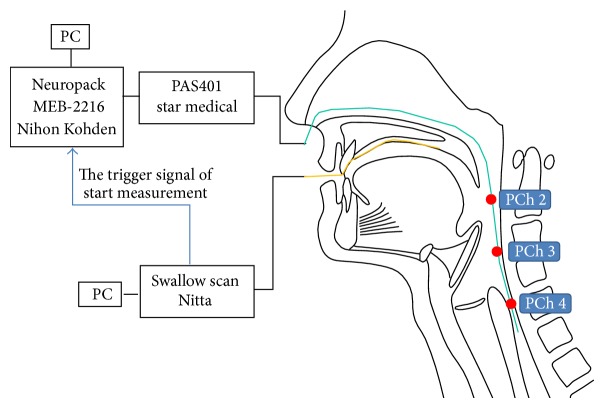
Simultaneous measurement system for tongue and pharyngeal pressures. PCh 1 was positioned approximately even with the nasopharynx, pharyngeal PCh 2 was placed approximately at the oropharynx, pharyngeal PCh 3 was placed approximately at the hypopharynx, and pharyngeal PCh 4 was positioned at the upper border of the high-pressure zone of the UES.

Tongue pressures and pharyngeal pressures were recorded synchronously during dry swallowing. To synchronize data, the trigger signal to start measurement from the swallow scan was sent to the Neuropack MEB-2216, and the tongue pressure was measured at the same time (Figure 1). The data of tongue pressures and pharyngeal pressures were recorded and analyzed on a separate PC. To confirm synchronization between the data, one of the measuring points of the sensor sheet was placed on a manometer, and loading was provided 15 times by the examiner's finger. The mean discrepancy between the output timing of both sensors was 4.67 ± 4.81 msec. The discrepancy was so negligible small that the data of two sources were confirmed to be synchronized.

**Figure 2:**
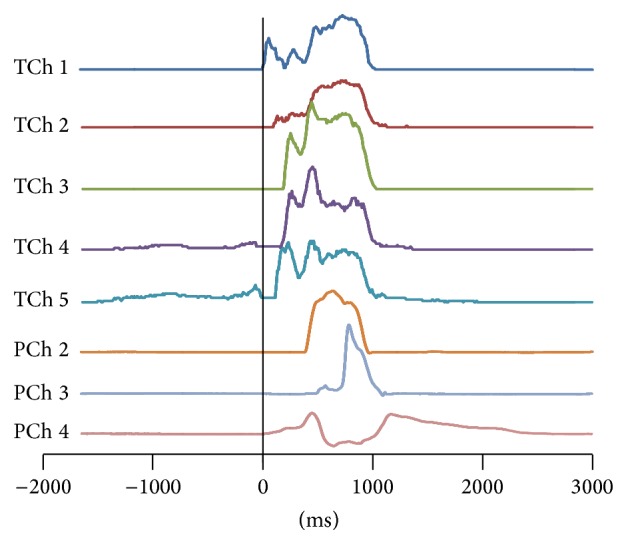
Typical waves of simultaneous recording of tongue (TChs 1–5) and pharyngeal (PChs 2–4) pressures.

**Figure 3:**
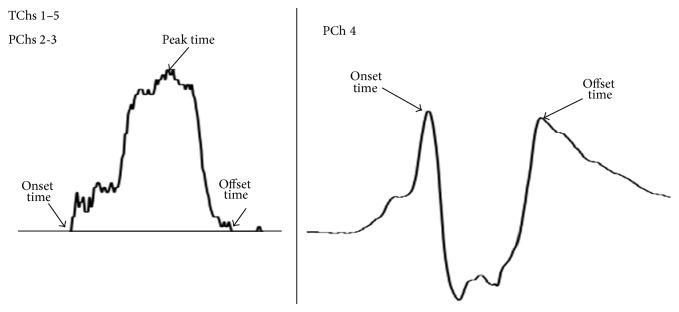
Analyzed parameters on the raw wave forms from the sensor sheet (TChs 1–5) and pharyngeal manometry (PChs 2–4).

Figure 2 shows typical waves of a simultaneous recording of tongue (TChs 1–5) and pharyngeal (PChs 2–4) pressures. Because the most proximal pharyngeal sensor, PCh 1, was highly variable individually in timing at peak pressure, it was excluded from the analysis in this study. The timings of onset, peak, and offset of tongue and pharyngeal pressures were measured on the waveform of each recording offline (Figure 3). Since PCh 4 was positioned at the upper portion of the UES, onset was defined as the timing at the highest pressure just before the pressure drop due to UES relaxation. The offset of PCh 4 was defined as the timing at the highest pressure just after the pressure drop.

### 2.3. Tongue Pressure Measurement

**Figure 4:**
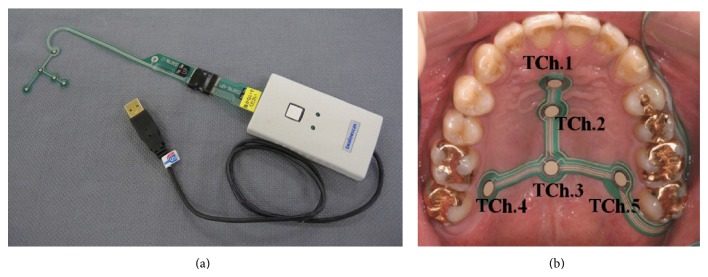
Swallow scan system and location of sensing points: (a) swallow scan system and sensor sheet and (b) intraoral view of attached sensor sheet and location of sensing points. TCh 1 was set at the anterior-median region, TCh 2 was set at the mid-median region, TCh 3 was set at the posterior-median region, TCh 4 was set at right circumferential region, and TCh 5 was situated at the left circumferential region of the hard palate.

The tactile sensor system swallow scan (Nitta, Tokyo, Japan) with a special sensor sheet for measuring tongue pressure was used in this study (Figure 4) [6]. The thickness of the sensor sheet was about 0.1 mm, and it had five measuring points. Three measuring points (TChs 1–3) were placed along the median line (TCh 1 was set at the anterior-median region, TCh 2 was set at the mid-median region, and TCh 3 was set at the posterior-median region), and two sensors (TChs 4 and 5) were situated in the posterior-circumferential regions of the hard palate. A small, medium, or large sensor sheet was selected for each subject according to the size of the hard palate. Before recording, the sensor sheet was attached to the palatal surface of the palatal mucosa directly with a sheet-type denture adhesive (Touch Correct II; Shionogi, Osaka, Japan). The wire was then connected to the computer exiting the oral cavity via the oral vestibule to avoid interference with the occlusion. After attaching the sensor sheet to the palate, calibration was performed by applying negative pressure on the cable of the sensor sheet using a vacuum pump. The pressure measured by the sensors was thus transmitted in real time to a personal computer, where the data were displayed and saved at 100 Hz.

### 2.4. Pharyngeal Manometry

A manometric catheter (P604-OSH-1, Star Medical, Tokyo, Japan) with four pharyngeal pressure sensors was inserted transnasally, through the pharynx, and into the proximal esophagus. Adjacent sensors were 3 cm apart. The manometry catheter was 130 cm long, 6 Fr (2 mm) in diameter. Correct catheter placement was determined by confirming that the most distal sensor was in the high-pressure zone of the UES at rest and an M-wave was clearly observed on dry swallowing. The catheter was then secured to the facial buccal region with surgical tape to minimize sensor movement. The sensors were thus placed in the following pharyngeal locations: pharyngeal Ch 1 (PCh 1; most proximal) was positioned approximately even with the nasopharynx, pharyngeal Ch 2 (PCh 2) was placed approximately at the oropharynx, pharyngeal Ch 3 (PCh 3) was placed approximately at the hypopharynx, and pharyngeal Ch 4 (PCh 4; most distal) was positioned at the upper border of the high-pressure zone of the UES. The pressures measured by the manometry were amplified (PAS401, Star Medical) and input into the electrode junction box (Neuropack MEB-2216, Nihon Kohden, Tokyo, Japan). The pharyngeal pressure was measured with the sampling frequency at 1000 Hz.

### 2.5. Data Analysis

To evaluate the temporal relationships of tongue pressure and pharyngeal pressure, and between tongue pressure and pharyngeal pressure, the differences in the order of onset, peak, and offset at each channel were examined after setting the onset of TCh 1 as 0 sec. In the analyses, uniformity of variance was determined by a Kolmogorov-Smirnov test. When uniform variance was found, significant differences were determined by one-way analysis of variance (ANOVA) and Tukey's post hoc test. The interclass correlation coefficient was used to evaluate the correlations between events at each TCh and PCh for describing the coordination of tongue and pharyngeal pressure production. Statistical analysis was performed using IBM SPSS Statistics Version 19 (IBM Japan, Tokyo, Japan), and statistical significance was set at *P* < 0.05.

## 3. Results

### 3.1. Time Sequence for Tongue and Pharyngeal Pressures

**Figure 5:**
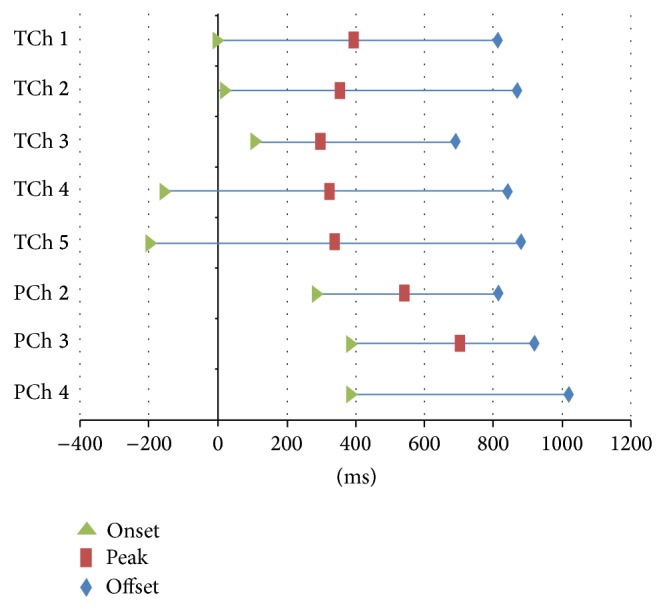
Sequential order of tongue and pharyngeal pressures during dry swallowing. Time “0” was set at the onset of TCh 1.

Figure 5 and Table 1 show the time sequences for tongue pressure at the five channels and pharyngeal pressure at the three channels during dry swallowing, where time “0” was set at the onset of tongue pressure at Ch 1.  Table 2 shows the comparison of the time sequence between the time events on tongue and pharyngeal pressures.

When comparing the tongue pressures produced during dry swallowing among TChs 1–5, the onset times at the posterior-lateral parts of the hard palate (TChs 4 and 5) were significantly earlier than that at the mid- and posterior-median parts (TChs 2 and 3), and the offset time was significantly earlier at Ch 3 than at Chs 2 and 5. There was no significant sequential difference in peak time of TChs 1–5.

When comparing the pharyngeal pressures produced in PChs 2–4, the offset time at mid-pharynx (PCh 2) was significantly earlier than that at the UES (PCh 4). There was no significant difference among the onset times at PChs 2–4 (*P* > 0.10). The peak time at PCh 2 tended to be earlier than that at the lower-pharynx (PCh 3), but not significantly (*P* = 0.066).

When comparing the tongue pressure with the pharyngeal pressure produced during dry swallowing, the onset time was earlier at TChs 1–5 than at PChs 2–4. The peak time of tongue pressure at the anterior-median part of the hard palate (TCh 1) was earlier than at the lower-pharynx (PCh 3), that at TChs 2–5 was earlier than at the mid- and lower-pharynx (PChs 2 and 3), and that at TChs 1–5 was earlier than the offset time at PChs 2–4. The offset time was later at TChs 1–5 than the onset time at PChs 2–4, that at TChs 1, 2, 4, and 5 was later than the peak time of midpharyngeal pressure (PCh 2), that at TChs 2 and 5 was later than the peak time of lower-pharyngeal pressure (PCh 3), that at TCh 3 was earlier than the offset time of lower-pharyngeal pressure (PCh 3), and that at TChs 1, 3, and 4 was earlier than the offset time of pharyngeal pressure at the UES (PCh 4).

### 3.2. Relationships between Events of Tongue and Pharyngeal Pressure

The interclass correlation coefficients between events of tongue and pharyngeal pressures are shown in Table 3. The onset time of pharyngeal pressure was not correlated with the onset time and the offset time of tongue pressure. However, the onset time of PChs 2–4 was temporally time-locked to the peak time of TChs 1–5, and there was an especially strong relationship between PChs 2–4 and TCh 3 (posterior-median part of the hard palate). The peak time of midpharyngeal pressure (PCh 2) had no significant correlation with the onset time of tongue pressure, but it had significant correlations with the peak time and the offset time of each TCh, except for the offset time at TCh 2. The peak time of lower-pharyngeal pressure (PCh 3) had no significant correlations with the onset time and the peak time of tongue pressure, but it had significant correlations with the offset time of TChs 1–5. The offset time of PChs 2–4 had no significant correlations with the onset time and peak time of tongue pressure but was temporally time-locked to the offset time of TChs 1–5.

## 4. Discussion

In research conducted to date on the mechanisms involved in bolus propulsion from the oral cavity to the pharynx and from there to the esophagus, it has been difficult to evaluate the tongue and the deep muscles of the pharynx activated during swallowing using EMG, so VF has been used in most of the research. More recently, there has been some studies involving swallowing dynamics using MRI and CT [30–34], but because of the large equipment required and the high cost involved, only a limited number of facilities is able to conduct these kinds of studies. Moreover, the analysis requires significant expertise and is time consuming, and CT scans are invasive because of the radiation involved. Given these considerations, attempts have been made to measure the intraluminal pressure from the oral cavity to the pharynx [35–37], but because of technical limitations, it has not been possible to identify clear temporal coordination in any of these.

In the present study, simultaneous measurements were conducted using a pressure sensor that was placed in the oral cavity that sensed pressure at five locations, and manometry was performed at three locations in the pharynx. This produced clear images of the pressure generated from the oral cavity to the pharynx during dry swallowing, the first time that such images have been produced on a time axis. The results are significant in terms of understanding the biomechanical coordination of the various organs during swallowing.

In the present study, because sensors were placed in both the oral cavity and at the pharynx, there is a possibility that they may have affected swallowing function. With respect to the tongue pressure sensors, it has been reported that placement in the oral cavity did not affect swallowing function [7]. In the present study, the onset phase of recorded tongue pressure when swallowing saliva was in common with those reported by Hori et al. [38] and Furuya et al. [39]. Specifically, the onset time of tongue pressure when swallowing saliva began when the tongue came in contact with the posterior-circumferential part of the hard palate (TChs 4-5), and contact then moved to the anterior-median part of the hard palate and finally to the posterior part of the hard palate, and for all of the tongue pressures when swallowing saliva, the TCh values reached the peak time largely simultaneously.

At the same time, however, it has been reported that placement of small-diameter manometers in the pharynx does not have a major effect on swallowing dynamics [40, 41]. The diameter of the manometers used in the present study was 6 Fr, which was used because it is the smallest available [42], and it is possible that it does not affect swallowing dynamics. It is possible to determine swallowing propagation speed by measuring the pharyngeal pressure. The propagation speed of the pharyngeal pressure peak time of the middle to lower pharyngeal region (PCh 2-PCh 3) obtained in the present study was 18.62 cm/sec (3 cm between manometry sensors, PCh 3 peak time mean value 700.86 msec, PCh 2 peak time mean value 538.71 msec), which is close to the value of 20.7 cm/sec measured by Mori [43] using conventional manometry. Based on the above, we believe that the system for simultaneous measurement of tongue pressure and pharyngeal pressure used in the present study is appropriate in terms of measuring physiological swallowing dynamics.

The results of the present study indicate that after all of the TChs had appeared, all of the PChs onset times appeared, and it was confirmed that the pressure onset occurred because there was a difference between the oral cavity and the pharynx in the times at which the food bolus was sent. Additionally, it was found that there were no differences in the onset of all TChs peak times and all PChs onset times. Based on these findings, when the tongue pressure in the oral cavity reached the peak time, that is, immediately after the propulsion force applied to the food bolus in the oral cavity reached its maximum level, it was found that pharyngeal pressure began to appear in preparation for the intake of the food bolus coming from the oral cavity. There was a certain difference in the peak times at which the transition from PCh 2 to PCh 3 occurred after the TChs peak time was reached, but it was not significant. This time lag between the peak time of the oral cavity and that of the pharynx and also the time lag between the oropharyngeal peak time and the hypopharyngeal peak time caused the maximum propulsion force of the food bolus produced in the oral cavity to be maintained as the food bolus continued on to the pharynx, and it is believed that this propulsion force efficiently sends the food bolus from above the pharynx to below it. Moreover, despite the fact that a significant difference was seen in the onset times between the tongue pressure and the pharyngeal pressure, the difference in the offset times was not all that significant. This suggests that positive pressure is maintained from the oral cavity to the pharynx during the time that the food bolus is being sent, so that it does not proceed in the reverse direction before swallowing has been completed.

In the present study, the interclass coefficient correlation was used to investigate the temporal relationship between the tongue pressure and the pharyngeal pressure. The interclass coefficient correlation indicates the degree of mutual coordination between the changes of two time-based events; so, for example, if the interclass coefficient correlation between event A and event B is 1 and the timing of event A is doubled (delayed), the timing of event B will be similarly doubled (delayed).

First, the significant correlations between the peak time of the tongue pressure for TCh 3 and the onset and peak times of the various pharyngeal pressures suggest that maximal closure of the posterior median part of the hard palate, which is nearest to the pharyngeal side in the oral cavity, is closely related to the process that forms the maximum pharyngeal pressure. TCh 3 is positioned at the posterior median part of the hard palate, and the tongue pressure that occurs at this site is relatively small and short when swallowing saliva and when swallowing liquids [6, 38]. However, this is an important site in terms of sending the food bolus, which includes the food bolus between the dorsal median region of the tongue and the palate, from the oral cavity to the pharynx. Hori et al. [7] conducted simultaneous measurements of the tongue pressure and VF during swallowing of liquids and reported an interclass correlation coefficient with the peak timing of the tongue pressure for TCh 3 at the midpoint (0.508) of the timing at which the hyoid bone was farthest upward and forward. The coordination between the peak of the pressure at posterior site of the hard palate and the onset of pharyngeal pressure may suggest that this is important in terms of a smooth transition of the food bolus from the oral phase to the pharyngeal phase of swallowing. Furthermore, the existence of a strong correlation between the offset time of the tongue pressure for TCh and the offset time of the pharyngeal pressure in PChs 2–4 suggests that the contact between the tongue and the palate acts as a functional anchor for the hard palate in order to maintain pharyngeal pressure [44]. Hori et al. [7] also reported a medium to strong correlation (0.496–0.827) between the offset timing of the tongue pressure and the timing at which the hyoid bone was farthest upward and forward.

Thus, the outcome of the present study clearly shows a rational coordination in the time-based onset phase of tongue pressure and pharyngeal pressure in the voluntary swallowing of saliva. This coordination could serve as a reference in the noninvasive evaluation of swallowing movements in the future, providing a means for detecting swallowing impairments. For example, if the onset time of the pharyngeal pressure does not appear even though the tongue pressure has reached its peak time, the food bolus, for which the propulsion force is at its maximum in the oral cavity, could be propelled too vigorously toward the pharynx, causing predeglutitive aspiration. Conversely, if there is a difference between the offset time of the tongue pressure and the offset time of the pharyngeal pressure, so that the tongue pressure reaches its offset time earlier than the pharyngeal pressure, positive pressure in the oral cavity could disappear, creating a difference between the oral cavity and pharyngeal pressures. This could cause pharyngeal residue or induce the food bolus to move in the reverse direction, from the pharynx to the oral cavity. In the future, simultaneous measurements of the tongue pressure and pharyngeal pressure need to be conducted in patients with swallowing impairments, and these findings need to be verified.

A limitation of this study was the fact that dry swallowing was used, without a food bolus. Previous studies have reported that tongue pressure and pharyngeal pressure are different when saliva is swallowed and when a liquid is swallowed [19, 20, 22, 38]. Given that, the authors intend to conduct further analyses using a variety of food boluses in the future. When doing those analyses, we believe that the onset time, peak time, and offset time parameters, which are detailed time-based parameters that were used in the present study, will be effective in evaluating modulations in pressure production.

## Figures and Tables

**Table 1:** Onset, peak, and offset times (mean and SD) of tongue and pharyngeal pressures.

	TCh 1	TCh 2	TCh 3	TCh 4	TCh 5	PCh 2	PCh 3	PCh 4
Onset time								
Mean	0.00	21.59	108.80	−153.86	−194.08	286.57	387.80	387.49
(SD)	(0.00)	(188.63)	(182.55)	(310.37)	(339.43)	(221.19)	(234.99)	(247.17)
Peak time								
Mean	392.24	352.22	296.31	322.43	338.14	538.71	700.86	
(SD)	(276.35)	(223.36)	(249.01)	(272.17)	(275.62)	(274.76)	(243.08)	
Offset time								
Mean	810.39	869.14	687.71	841.84	882.24	814.04	918.33	1016.98
(SD)	(273.94)	(353.04)	(371.42)	(432.29)	(451.21)	(378.79)	(275.15)	(234.96)

[msec].

**Table 2:** Comparison of the time sequence between time events of tongue and pharyngeal pressures.

	Onset time					Peak time					Offset time				
	TCh 1	TCh 2	TCh 3	TCh 4	TCh 5	TCh 1	TCh 2	TCh 3	TCh 4	TCh 5	TCh 1	TCh 2	TCh 3	TCh 4	TCh 5
Onset Time															
PCh 2		−0.000	−0.021	−0.000	−0.000	0.782	0.999	1.000	1.000	1.000	+0.000	+0.000	+0.000	+0.000	+0.000
PCh 3		−0.000	−0.000	−0.000	−0.000	1.000	1.000	0.932	0.999	1.000	+0.000	+0.000	+0.000	+0.000	+0.000
PCh 4		−0.000	−0.000	−0.000	−0.000	1.000	1.000	0.934	0.999	1.000	+0.000	+0.000	+0.000	+0.000	+0.000
Peak time															
PCh 2		−0.000	−0.000	−0.000	−0.000	0.173	−0.010	−0.000	−0.001	−0.003	+0.000	+0.000	0.150	+0.000	+0.000
PCh 3		−0.000	−0.000	−0.000	−0.000	−0.000	−0.000	−0.000	−0.000	−0.000	0.725	+0.043	1.000	0.232	+0.016
Offset time															
PCh 2		−0.000	−0.000	−0.000	−0.000	−0.000	−0.000	−0.000	−0.000	−0.000	1.000	1.000	0.443	1.000	0.998
PCh 3		−0.000	−0.000	−0.000	−0.000	−0.000	−0.000	−0.000	−0.000	−0.000	0.749	1.000	−0.000	0.990	1.000
PCh 4		−0.000	−0.000	−0.000	−0.000	−0.000	−0.000	−0.000	−0.000	−0.000	−0.002	0.160	−0.000	−0.026	0.313

−: indicates that the event of tongue pressure was significantly earlier than that of pharyngeal pressure (*P* < 0.05).

+: indicates that the event of tongue pressure was significantly later than that of pharyngeal pressure (*P* < 0.05).

*P* value showed ANOVA and Tukey's post hoc test for comparison of the time sequence between time events of tongue and pharyngeal pressures.

**Table 3:** Interclass correlation coefficients of time sequences between tongue and pharyngeal pressures.

	Onset time					Peak time					Offset time				
	TCh 1	TCh 2	TCh 3	TCh 4	TCh 5	TCh 1	TCh 2	TCh 3	TCh 4	TCh 5	TCh 1	TCh 2	TCh 3	TCh 4	TCh 5
Onset time															
PCh 2	−0.453	0.112	0.321	−0.244	−0.273	0.645^**^	0.750^**^	0.943^**^	0.667^**^	0.478^**^	−0.109	−0.268	0.136	−0.089	−0.094
PCh 3	−0.575	−0.049	0.050	−0.383	−0.396	0.589^**^	0.671^**^	0.783^**^	0.553^**^	0.405^**^	0.052	−0.190	0.200	−0.015	0.002
PCh 4	−0.549	−0.176	0.151	−0.287	−0.317	0.505^**^	0.522^**^	0.791^**^	0.598^**^	0.278^*^	0.051	−0.115	0.286	−0.010	−0.030
Peak time															
PCh 2	−0.657	−0.274	−0.180	−0.483	−0.497	0.565^**^	0.464^**^	0.579^**^	0.443^**^	0.281^*^	0.443^**^	0.059	0.520^**^	0.254^*^	0.269^*^
PCh 3	−0.806	−0.558	−0.488	−0.627	−0.632	0.209	0.089	0.148	0.050	−0.044	0.784^**^	0.370^**^	0.680^**^	0.433^**^	0.435^**^
Offset time															
PCh 2	−0.697	−0.544	−0.463	−0.588	−0.594	0.009	−0.0132	−0.026	−0.084	−0.166	0.580^**^	0.302^*^	0.484^**^	0.385^**^	0.416^**^
PCh 3	−0.848	−0.684	−0.640	−0.719	−0.713	−0.152	−0.257	−0.215	−0.290	−0.319	0.837^**^	0.548^**^	0.542^**^	0.536^**^	0.574^**^
PCh 4	−0.903	−0.763	−0.732	−0.794	−0.791	−0.355	−0.461	−0.416	−0.461	−0.501	0.592^**^	0.390^**^	0.275^*^	0.408^**^	0.504^**^

^*^ *P* < 0.05, ^**^ *P* < 0.01.

## References

[1] Leopold N. A., Kagel M. (1983). Swallowing, ingestion and dysphagia: a reappraisal. *Archives of Physical Medicine and Rehabilitation*.

[2] Leopold N. A., Kagel M. C. (1997). Dysphagia—ingestion or deglutition? A proposed paradigm. *Dysphagia*.

[3] Okada T., Aoyagi Y., Inamoto Y., Saitoh E., Kagaya H., Shibata S., Ota K., Ueda K. (2013). Dynamic change in hyoid muscle length associated with trajectory of hyoid bone during swallowing: analysis using 320-row area detector computed tomography. *Journal of Applied Physiology*.

[4] Ono T., Hori K., Nokubi T. (2004). Pattern of tongue pressure on hard palate during swallowing. *Dysphagia*.

[5] Ono T., Hori K., Masuda Y., Hayashi T. (2010). Recent advances in sensing oropharyngeal swallowing function in Japan. *Sensors*.

[6] Hori K., Ono T., Tamine K.-I., Kondo J., Hamanaka S., Maeda Y., Dong J., Hatsuda M. (2009). Newly developed sensor sheet for measuring tongue pressure during swallowing. *Journal of Prosthodontic Research*.

[7] Hori K., Taniguchi H., Hayashi H. (2013). Role of tongue pressure production in oropharyngeal swallow biomechanics. *Physiological Reports*.

[8] Kahrilas P. J., Lin S., Logemann J. A., Ergun G. A., Facchini F. (1993). Deglutitive tongue action: volume accommodation and bolus propulsion. *Gastroenterology*.

[9] Hara M., Takasaki K., Mastuo K. (2012). Dynamics of the swallowing pressure in normal volunteers evaluated by high-resolution manometry. *Deglutition*.

[10] Kobayashi H., Sekizawa K., Sasaki H. (1997). Aging effects on swallowing reflex. *Chest*.

[11] Logemann J. A. (1990). Effects of aging on the swallowing mechanism. *Otolaryngologic Clinics of North America*.

[12] Tracy J. F., Logemann J. A., Kahrilas P. J., Jacob P., Kobara M., Krugler C. (1989). Preliminary observations on the effects of age on oropharyngeal deglutition. *Dysphagia*.

[13] Robbins J., Hamilton J. W., Lof G. L., Kempster G. B. (1992). Oropharyngeal swallowing in normal adults of different ages. *Gastroenterology*.

[14] Cook I. J., Dodds W. J., Dantas R. O., Kern M. K., Massey B. T., Shaker R., Hogan W. J. (1989). Timing of videofluoroscopic, manometric events, and bolus transit during the oral and pharyngeal phases of swallowing. *Dysphagia*.

[15] Logemann J. A. (1993). *Manual for the Videofluorographic Study of Swallowing*.

[16] Muray J. (1999). *Manual of Dysphagia Assessment in Adults*.

[17] Dantas R. O., Kern M. K., Massey B. T. (1990). Effect of swallowed bolus variables on oral and pharyngeal phases of swallowing. *American Journal of Physiology: Gastrointestinal and Liver Physiology*.

[18] Kahrilas P. J., Logemann J. A., Lin S., Ergun G. A. (1992). Pharyngeal clearance during swallowing: a combined manometric and videofluoroscopic study. *Gastroenterology*.

[19] Perlman A. L., Schultz J. G., VanDaele D. J. (1993). Effects of age, gender, bolus volume, and bolus viscosity on oropharyngeal pressure during swallowing. *Journal of Applied Physiology*.

[20] Shaker R., Ren J., Podvrsan B., Dodds W. J., Hogan W. J., Kern M., Hoffmann R., Hintz J. (1993). Effect of aging and bolus variables on pharyngeal and upper esophageal sphincter motor function. *American Journal of Physiology*.

[21] Gumbley F., Huckabee M. L., Doeltgen S. H., Witte U., Moran C. (2008). Effects of bolus volume on pharyngeal contact pressure during normal swallowing. *Dysphagia*.

[22] Butler S. G., Stuart A., Castell D., Russell G. B., Koch K., Kemp S. (2009). Effects of age, gender, bolus condition, viscosity, and volume on pharyngeal and upper esophageal sphincter pressure and temporal measurements during swallowing. *Journal of Speech, Language, and Hearing Research*.

[23] Nativ-Zeltzer N., Kahrilas P. J., Logemann J. A. (2012). Manofluorography in the evaluation of oropharyngeal dysphagia. *Dysphagia*.

[24] Nekl C. G., Lintzenich C. R., Leng X., Lever T., Butler S. G. (2012). Effects of effortful swallow on esophageal function in healthy adults. *Neurogastroenterology & Motility*.

[25] Ono T., Kumakura I., Arimoto M., Hori K., Dong J., Iwata H., Nokubi T., Tsuga K., Akagawa Y. (2007). Influence of bite force and tongue pressure on oro-pharyngeal residue in the elderly. *Gerodontology*.

[26] Stierwalt J. A., Youmans S. R. (2007). Tongue measures in individuals with normal and impaired swallowing. *American Journal of Speech-Language Pathology*.

[27] Robbins J., Kays S. A., Gangnon R. E., Hind J. A., Hewitt A. L., Gentry L. R., Taylor A. J. (2007). The effects of lingual exercise in stroke patients with dysphagia. *Archives of Physical Medicine and Rehabilitation*.

[28] Hori K., Ono T., Iwata H., Nokubi T., Kumakura I. (2005). Tongue pressure against hard palate during swallowing in post-stroke patients. *Gerodontology*.

[29] Konaka K., Kondo J., Hirota N., Tamine K., Hori K., Ono T., Maeda Y., Sakoda S., Naritomi H. (2010). Relationship between tongue pressure and dysphagia in stroke patients. *European Neurology*.

[30] Anagnostara A., Stoeckli S., Weber O. M., Kollias S. S. (2001). Evaluation of the anatomical and functional properties of deglutition with various kinetic high-speed MRI sequences. *Journal of Magnetic Resonance Imaging*.

[31] Beer A. J., Hellerhoff P., Zimmermann A., Mady K., Sader R., Rummeny E. J., Hannig C. (2004). Dynamic near-real-time magnetic resonance imaging for analyzing the velopharyngeal closure in comparison with videofluoroscopy. *Journal of Magnetic Resonance Imaging*.

[32] Hartl D. M., Albiter M., Kolb F., Luboinski B., Sigal R. (2003). Morphologic parameters of normal swallowing events using single-shot fast spin echo dynamic MRI. *Dysphagia*.

[33] Inamoto Y., Fujii N., Saitoh E., Baba M., Okada S., Katada K., Ozeki Y., Kanamori D., Palmer J. B. (2011). Evaluation of swallowing using 320-detector-row multislice CT. Part II: kinematic analysis of laryngeal closure during normal swallowing. *Dysphagia*.

[34] Okada T., Aoyagi Y., Saitoh E., Inamoto Y., Kagaya H., Shibata S., Ota K., Ueda K. (2013). Dynamic change in hyoid muscle length associated with trajectory of hyoid bone during swallowing: analysis using 320-row area detector computed tomography. *Journal of Applied Physiology*.

[35] Tanaka E., Ono Y., Gonda Y. (2001). Analysis of mandibular movement, and oral and pharyngeal pressure during deglutition. *Journal of Osaka Dental University*.

[36] Arioka K., Ishida R., Yanagi Y. (2005). Simultaneous analysis of the pressure of the tongue and pharyngeal during swallowing. *The Journal of Okayama Dental Society*.

[37] Uesugi N., Ono Y., Komasa Y. (2008). Influence of postural changes on the dynamics of swallowing. *Osaka Odontological Society*.

[38] Hori K., Tamine K., Barbezat C., Maeda Y., Yamori M., Müller F., Ono T. (2011). Influence of chin-down posture on tongue pressure during dry swallow and bolus swallows in healthy subjects. *Dysphagia*.

[39] Furuya J., Nakamura S., Ono T., Suzuki T. (2012). Tongue pressure production while swallowing water and pudding and during dry swallow using a sensor sheet system. *Journal of Oral Rehabilitation*.

[40] Lydon S. B., Dodds W. J., Hogan W. J., Arndorfer R. C. (1975). The effect of manometric assembly diameter on intraluminal esophageal pressure recording. *The American Journal of Digestive Diseases*.

[41] Cook I. J., Dent J., Collins S. M. (1989). Upper esophageal sphincter tone and reactivity to stress in patients with a history of globus sensation. *Digestive Diseases and Sciences*.

[42] Salassa J. R., DeVault K. R., McConnel F. M. (1998). Proposed catheter standards for pharyngeal manofluorography (videomanometry). *Dysphagia*.

[43] Mori T. (1989). Conduction velocity of swallowing pressures in the pharynx and the cervical esophagus. *Practica Oto-Rhino-Laryngologica*.

[44] Steele C. M., Huckabee M. L. (2007). The influence of orolingual pressure on the timing of pharyngeal pressure events. *Dysphagia*.

